# Pediatric Epinephrine Auto-injector Accident Without Digital Ischemia

**DOI:** 10.7759/cureus.6435

**Published:** 2019-12-20

**Authors:** Alex L Shapiro, Danielle Ziehl

**Affiliations:** 1 Emergency Medicine, San Antonio Uniformed Services Health Education Consortium, Fort Sam Houston, USA

**Keywords:** epinephrine, digit, finger, ischemia

## Abstract

Accidental finger sticks with EpiPens and EpiPen Jr (epinephrine auto-injectors) are a very real hazard in those who carry them and their families. The most feared complications are digital ischemia and necrosis; however, long-term adverse effects are extremely rare. Treatment for a finger stick is controversial, ranging from intra-arterial inj­­ections of vasodilating agents to topical vasodilators to conservative management. We report a pediatric patient suffering from an EpiPen Jr accidental stick to the distal first digit who was successfully managed in a conservative fashion.

## Introduction

Epinephrine auto-injectors are common and continue to become more prevalent, increasing the chance of an accidental injection [1]. This is especially relevant with the increase in prophylactic prescriptions for pediatric auto-injectors in low patients. Although commonly seen as safe in adults, auto-injector data for pediatrics are more limited; there still persists the concern for digital ischemia and persistent paresthesias in the event of an accidental injection.

Epinephrine is the quintessential dual alpha and beta agonist; it has potent effects both peripherally on alpha adrenergic receptors as a vasoconstrictor and beta receptor effects on cardiac receptors as a chronotropic and ionotropic agent [2]. Beta receptors on the smooth muscle in the pulmonary vasculature help to relax and reverse some of the effects of anaphylaxis. Used locally, its vasoconstricting effects reduce the amount of local anesthetic needed for minor procedures as well as increase the duration of analgesic affect.

## Case presentation

**Figure 1:**
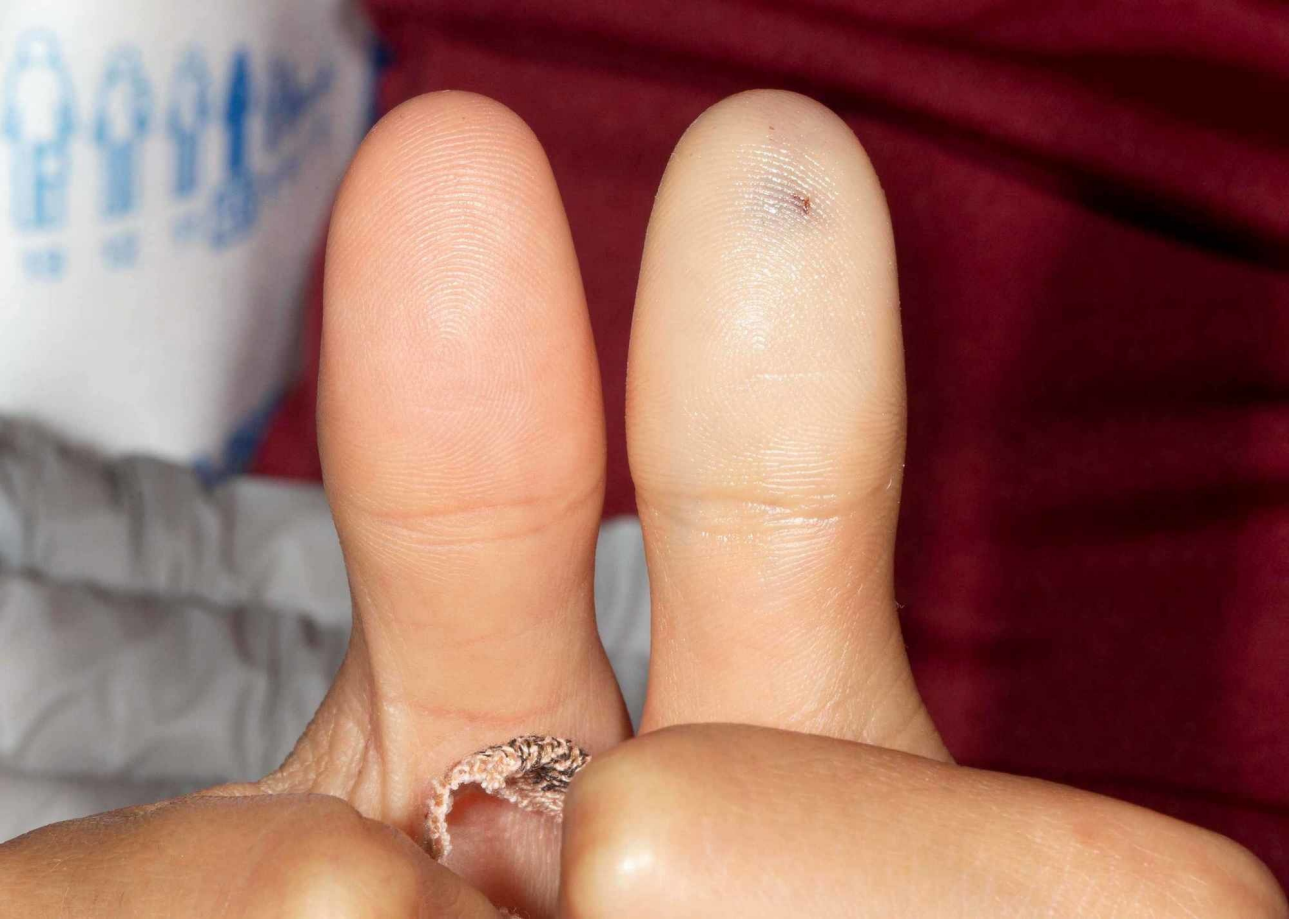
Unaffected and affected digits

A 12-year-old male presented to the emergency room within one hour of accidentally injecting his distal left thumb with his brother’s EpiPen Jr (epinephrine auto-injector, 0.15 mg). On arrival, his vitals were normal and the phalanx was blanched down to the thenar imminence. He complained of pain and decreased sensation from the distal finger to the thenar area. Capillary refill of the digit was unable to be obtained because the entire digit was blanched (see Figure 1). A plain film did not show evidence of retained needle or bone injury. Topical nitroglycerin was applied to the digit with warm packs placed around the hand and digit. After four hours, there was minimal improvement to the blanched digit and paresthesias. Otherwise, the patient was pain-free.

Orthopedics (hand) was consulted for a possible injection of subcutaneous medication for ischemia reversal. They did not feel an aggressive intervention was warranted and recommended continued observation. The patient was admitted to the pediatrics service, where he received frequent warm compresses and warm water baths to the affected digit. Symptoms resolved within an additional six hours; at that time there was no evidence of ischemia and the patient denied paresthesias, numbness, or pain. He was discharged without issue. 

## Discussion

Accidental digital injection with epinephrine has multiple case studies and case series demonstrating minimal long-term adverse effects [3,4]. This is found both in the emergency medicine literature and has been long accepted and known within the plastic surgery community [5,6]. The most common adverse reactions to digital epinephrine are paresthesias, all of which resolve by six months [4]. Epinephrine-induced digital ischemia has minimal long-term morbidity even with conservative management; aggressive treatments should generally be avoided given that the benefits rarely outweigh the risks. Historical treatments included subcutaneous injection of phentolamine (an alpha receptor antagonist) or terbutaline (a beta receptor agonist). Although rarely used, these reversal agents continue to be good alternatives if resolution of symptoms does not improve in a timely period and necrosis is suspected [7,8]. Less invasive methods include topical vasodilators, including topical nitroglycerin and a topical calcium channel blocker, both have been used but little data to demonstrate efficacy.

Other considerations with accidental digital injection with an epinephrine auto-injector include bone perforation. This is a rare occurrence, with very few case reports or other mentions. In the first cited case, Schintler and colleagues noted a clear bone perforation path on x-ray but without evidence of retained needle [9,10]. A concern for possible inoculation of the digit with bacteria was treated with a course of amoxicillin-clavulanic acid without complications. In the second cited case, a pediatric auto-injector was thought to perforate the femur at the correct location of administration; there was no needle retention nor long-term morbidity.

An extensive 2010 poison control data-oriented case series by Muck and colleagues demonstrates that, over six years, there were no significant long-term adverse effects associated to digital injection by epinephrine auto-injectors managed conservatively in adults [4]. Although this study did not include pediatric cases, it provides the most compelling evidence towards avoiding invasive and potentially risky procedures. Despite the lack of randomized controlled trials on the subject, non-invasive management of accidental digital injection seems to be safe.

The dogma that is persistently taught in medical schools about avoiding epinephrine administration in a digit should be re-evaluated based on re-appraisal of the literature. Plastic surgery has been using epinephrine judiciously in digits, and this has provided ample amount of evidence and literature as to its safety, as seen in multiple studies, totaling 4,221 cases that did not result in long-term digital ischemia [11,12]. This combined with case reports and case studies of benign outcomes of accidental digital injection with epinephrine auto-injectors can help minimize potentially harmful and non-efficacious interventions in the emergency room.

## Conclusions

There is a relative paucity of the literature reporting pediatric cases of epinephrine-induced digital ischemia. Although more research is needed, from our experience and that of others, the lessons learned in treating adults with epinephrine-induced digital ischemia can likely be safely applied to the pediatric population. An overall conservative approach is both safe and preferred.
